# PSPAB: Privacy-preserving average procurement bidding system with double-spending checking

**DOI:** 10.1371/journal.pone.0240548

**Published:** 2020-10-29

**Authors:** Li Li, Jiayong Liu, Peng Jia, Rongfeng Zheng

**Affiliations:** 1 College of Cybersecurity, Sichuan University, Chengdu, Sichuan, China; 2 School of Mathematics and Information Engineering, Chongqing University of Education, Chongqing, China; 3 College of Electronics and Information Engineering, Sichuan University, Chengdu, Sichuan, China; Wuhan University, CHINA

## Abstract

Several organizations use auctions in a procurement bidding system to maintain a low procurement cost. Although several privacy-preserving auction solutions for different application scenarios have been proposed over the past few decades, none of them can perform efficient average procurement bidding while ensuring strong privacy protection for the bids of suppliers. To address this problem, we propose PSPAB, a lightweight, secure average procurement bidding system based on cryptographic tools, to provide full privacy for bids. In addition, this system allows the procurement manager to identify the users in the case of double spending. We formally prove the security of PSPAB under a semi-honest adversary model. Experimental results validate the theoretical analysis and practical application of PSPAB in real-world scenarios.

## Introduction

Nowadays, government organizations and private businesses use various auction mechanisms for procuring goods and services. One of the most popular mechanisms is the standard lowest-price auction. The supplier who bids the lowest price is declared as the winner. However, the lowest-price auction mechanism has drawbacks. It can be expensive for the procurement manager if the winning supplier underestimates the cost of a project to seek the lowest price and eventually suffers from the well-known winner’s curse. For example, a supplier bids for a new construction project seeking to increase the winning probability as much as possible; hence, the supplier tends to submit as low a bid as possible and underestimates the eventual cost. As the project is underestimated, the winning supplier will risk defaulting on the project midway through completion to prevent a loss. Therefore, the average-bid auction is presented to overcome this problem [1]. The supplier with the closest bid to the average of all the submitted bids is declared as the winner. Our paper focuses on the average-bid auction procurement mechanism.

Over the past few decades, studies have been extensively conducted on the design of average-bid auction mechanisms [2–5], in which the procurement manager is assumed to be trustworthy and all the suppliers submit their bids unreservedly. However, in practice, the procurement manager and suppliers cannot be trusted unconditionally, as they might circumvent normal procurement to seek greater benefits. For example, a dishonest supplier may adapt their competing bid to win the auction by monitoring the bids of other suppliers. In addition, a faithless supplier may submit their bid more than once before the auction process to improve the winning probability. This operation will seriously disrupt the procurement process. Therefore, a privacy-preserving framework with double-spending checking should be designed for an average procurement bidding system.

However, two challenges must be overcome to design such a privacy-preserving average procurement bidding mechanism. The first challenge is how to design a secure average procurement bidding system to provide strong protection for the bids of suppliers without affecting the basic functions of the original system. A verifiable sealed-bid auction VSAB on the Ethereum blockchain was presented [6]. Subsequently, a collusion-resistant e-auction was implemented with a smart contract [7]. However, these two mechanisms do not provide privacy protection for bids during the entire procurement process, as the bids would be revealed eventually. To achieve full privacy for bids, secure design for double spectrum auctions (SDSA) was presented [8] to design an efficient and practical double spectrum auction. Furthermore, Wang et al. proposed a privacy-preserving and truthful double auction for a heterogeneous spectrum based on cryptographic primitives [9]. However, none of these studies provide the double-spending checking functionality.

The second challenge is how to design a solution to support the double-spending checking functionality. This functionality can handle the dishonest double-spending behaviors of suppliers. Nowadays, most studies that investigate the double-spending problem employ the blockchain technology. Several practical measures have been proposed [10–13] to address the double-spending problem in a blockchain. However, these solutions are strongly correlated to the blockchain property and are not easily scalable to other application scenarios. More similar to our application scenario, uCentive was proposed to allow users to earn and redeem unlinkable incentives from the provider [14]. Subsequently, a generic framework BBA was proposed to collect and accumulate incentives [15]. Although the two mechanisms support double-spending checking, they cannot identify the users in the case of double spending. Recently, a general framework has been presented to generate and accumulate incentives [16]. In this study, the provider could identify the double-spending users using a partially blind signature.

In summary, our contributions can be listed as follows.

- We propose a privacy-preserving average procurement bidding system for the first time. Compared with the existing mechanisms, PSPAB can provide strong privacy protection for the bids of suppliers throughout the procurement process. Privacy-preserving bid comparison has been achieved in our solution based on cryptographic tools, such as the Paillier cryptosystem and garbled circuits.
- To the best of our knowledge, our scheme is the first to provide a double-spending checking functionality for a procurement bidding system. This indicates that we can detect the double-spending behaviors of suppliers who intend to submit a bid more than once for improving their winning probability. This functionality allows us to identify double-spending suppliers.
- We formally prove that PSPAB is secure under a semi-honest adversary model through simulations. Moreover, we conduct extensive experiments to demonstrate the superior performance of our scheme in comparison with two state-of-the-art mechanisms. PSPAB has an acceptable computation time and communication overhead.

The rest of the paper is organized as follows. Section 2 overviews the related works. Section 3 presents the problem statement. Section 4 introduces the building blocks for PSPAB. The technical details of PSPAB are described in Section 5. The performance analysis and experimental results are given in Section 6 and Section 7, respectively. Section 8 concludes the whole paper.

## Related work

### Sealed price auction

Sealed-price auctions have been extensively investigated over the past few decades. A general secure auction system was presented [17] for secondary spectrum markets, in which the BGN cryptosystem was applied to achieve sealed-price comparison. However, the use of expensive bilinear pairing computation made the solution inefficient. More recently, Blass et al. designed a secure auction for blockchains based on the Fischlin tool [18]. However, this scheme had a high interactive communication overhead between the auctioneer and the suppliers. To achieve a higher efficiency, a fully private auction FPAHB for the highest bid was presented [19] to address the problem of sealed-bid comparison. However, FPAHB lacked feasibility for practical application, as a base value was required to be set in advance. In [20], the Paillier cryptosystem was employed to design a secure truthful double spectrum auction called PS-Trust. However, PS-Trust cannot protect the geo-location information privacy. Furthermore, by using the Paillier cryptosystem and garbled circuits, two privacy-preserving and truthful double auctions for spectrum allocation were proposed in different application scenarios [9, 21]. However, they both lack the ability to handle the double-spending problem.

### Double spending check

In [11], Rosenfeld et al. presented a hash-based proof of work to prevent double-spending currency. However, that study focused on blockchain currencies and it was infeasible for deployment in procurement bidding systems. In [10], a lightweight countermeasure that enabled the detection of double-spending attacks in fast transactions was proposed. For effective double-spending detection, a third-party “observer” or a deployment of “a listening period” was required, which resulted in an additional computation overhead. Everaere et al. proposed a risk management approach for double-spending protection by introducing the service of a trusted third-party trader [12]. However, the trusted third-party trader faced the unfairness problem with a high probability. Two generic frameworks for collecting and accumulate incentives were proposed [14, 15] by using different cryptographic tools. However, in these studies, double-spending solutions could only be used to deter users but such users could not be identified. Dimitriou et al. proposed REWARDS, a privacy-preserving rewards and incentive scheme for a smart electricity grid based on a partially blind signature, in which the double-spending users could be identified. However, REWARDS could not be applied to our scheme directly because it did not present the explicit algorithm to identify the double-spending suppliers.

For ease of explanation, Table 1 lists the comparisons with previous works.

**Table 1:** Comparisons with previous works.

Scheme	Identity verification	Double-spending check	Bid privacy
PS-TAHES [9]	×	×	√
SDSA [8]	×	×	√
Cream [7]	×	×	×
VSAB [6]	×	×	×
Strain [18]	×	×	√
PSPAB	√	√	√

## Problem statement

### System model

**Fig 1:**
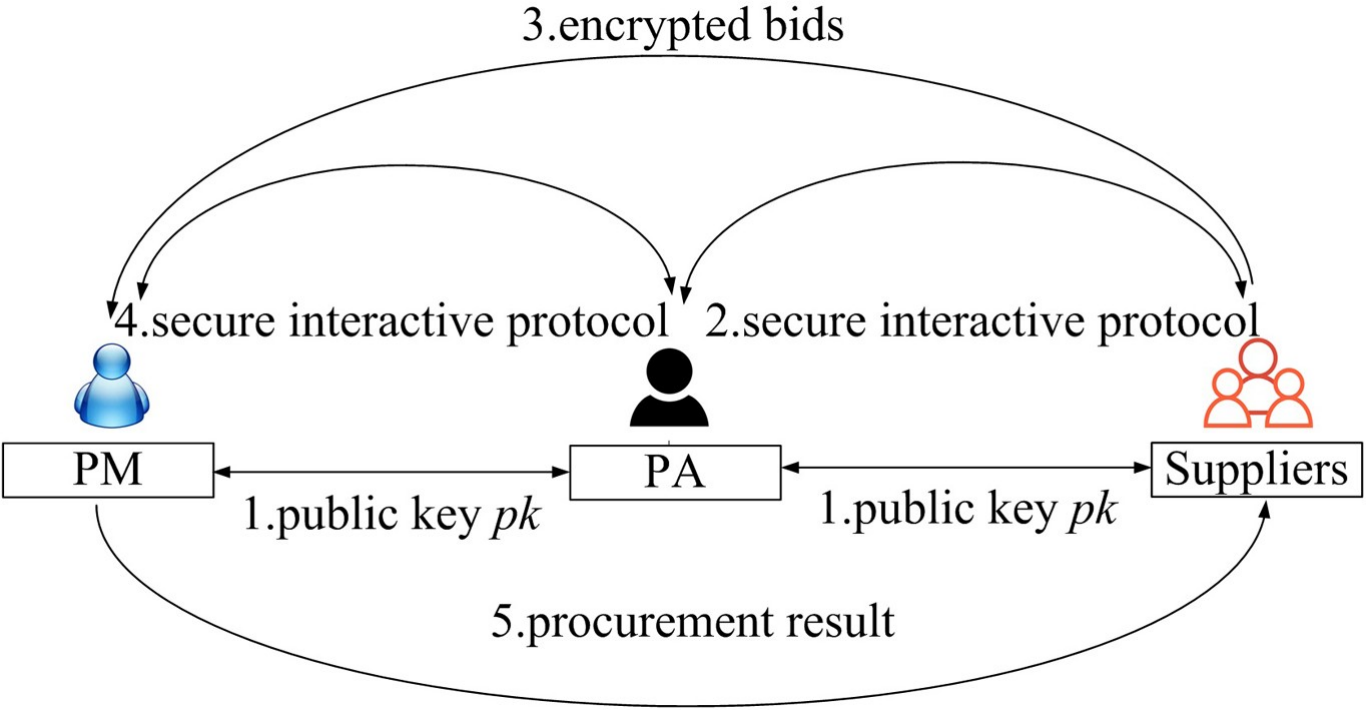
System model of PSPAB.

Our procurement bidding system is modeled as in Fig 1. It comprises three entities: suppliers, a procurement manager (PM), and a procurement agent (PA). The PM and the PA are semi-honest and do not collude with each other. The PA exists to cooperate with the PM to determine the winning supplier. The PA is transparent to the bids of suppliers and provides the critical security functionality in our system. Notably, such models involving agents are a popular trend in auction-based applications (e.g., [8, 9, 17]), and we adopt such a widely used model.

We consider an average procurement bidding system, where the PM finds the supplier with the bid closest to the average value of all the bids. In addition, our scheme supports the double-spending checking functionality. First, each supplier submits an identity commitment and proof to the PA, who verifies this proof. Subsequently, the suppliers submit their encrypted bids to the PM. Then, the PA cooperates with the PM to determine the winning supplier. During the process of procurement, the secret bid of each supplier is well protected. The mathematical notations throughout our paper are summarized in Table 2.

**Table 2:** Mathematical notations and semantic meanings.

Notations	Semantic meanings
*κ*	the security parameter
*p*, *q*	two large primes
*pk*_*i*_	the public key
*sk*_*i*_	the private key
*m*	the number of suppliers
*b*_*i*_	the *i*-th supplier’s bid
*b*	the average value of all the bids
〈*b*_*i*_〉	the encrypted bid of the *i*-th supplier under Pailier cryptosystem
$\hat{b}$	the garbled value in garbled circuits
*l*	the bit length of bids
*i*_*w*_	the index of the supplier with the bid closest to the average value *b*

### Threat model and design goals

According to the most popular security studies [8, 22, 23], we observe that the security threats are derived from the PM and the PA. Therefore, we consider the PM and the PA to be adversaries. The PM and the PA are assumed to be semi-honest and non-colluding. That is, the PM and the PA will faithfully obey the procurement rules but they want solicit secret bids beyond the procurement result.

Our objective in this study is to design a secure average procurement bidding mechanism with the double-spending checking functionality. In our scheme, the PM and the PA do not know the bid values. In other words, the scheme is provably secure under a standard security model and achieves information-theoretic security [24]. Additionally, we avoid using computation-extensive operations and adopt lightweight cryptographic operations to design a secure and efficient average procurement bidding system with an affordable computation time and communication cost. Furthermore, our scheme supports double-spending detection. Each supplier submits a commitment including identification information. Then, the PA verifies the identification proof.

Before presenting the formal security definition, we firstly introduce two basic terms that will be used in the following section.

- Computational indistinguishability. As represented in [25], a probability ensemble *Y* is defined as $Y={\left\{Y\left(x,\lambda \right)\right\}}_{x\in {\left(0,1\right)}^{*},\lambda \in N}$, where *x* is represented as the input and λ is represented as the security parameter. If two probability ensembles *Y* and *Z* are computationally indistinguishable, then *Y* and *Z* are denoted as *Y*≈_*c*_ *Z*. Then the following inequation holds, |*Pr*[*D*(*Y*(*x*, λ))] = 1|−|*Pr*[*D*(*Z*(*x*, λ))] = 1|≤*μ*(λ), where *D* denotes a non-uniform polynomial time algorithm and *μ*(⋅) denotes a negligible function.
- Composition theorem for semi-honest model. As described in [26], we suppose that a functionality *f*_2_ is privately reducible to another functionality *f*_1_. If there exists an algorithm for privately computing *f*_1_, then there exists an algorithm for privately computing *f*_2_.

## Building blocks

In this section, we introduce building blocks for PSPAB, including Paillier encryption, zero-knowledge proof, garbled circuits, and partially blinded signature.

### Paillier encryption

Based on the hardness of the composite residuosity class problem, Paillier cryptosystem with the homomorphic property was proposed in [27]. First, two large prime integers *p* and *q* are selected. Let *n* = *p* ⋅ *q*, where *Z*_*n*^2^_ is the set of integers modulo *n*^2^ and $Z_{n^{2}}^{*}$ is a subset of *Z*_*n*^2^_ relatively prime to *n*^2^. We select a random value $g\overset{\$}{\leftarrow }Z_{n^{2}}^{*}$ and compute $\lambda =\left(p-1\right)\left(q-1\right),k=\frac{\left(g^{\lambda }\mathrm{mod}n^{2}-1\right)}{n},\xi =k^{-1}\mathrm{mod}n$, where λ is the least common multiple. Then the public key is *pk* = (*g*, *n*) and the private key is *sk* = λ. Let *m* ∈ *Z*_*n*_ denote as the plaintext. We select $r\overset{\$}{\leftarrow }Z_{n}^{*}$ and compute the ciphertext *c* ∈ *Z*_*n*^2^_ as *c* = *E*(*m*, *r*) = *g*^*m*^ *r*^*n*^ mod *n*^2^. Finally the decryption can be implemented by $D\left(E\left(m,r\right)\right)=m=\frac{c^{\lambda }\mathrm{mod}n^{2}-1}{n}\xi \mathrm{mod}n$. Paillier cryptosystem has the following homomorphic properties.

$$\begin{array}{c} \begin{array}{cc} & D\left(E\left(m_{1},r_{1}\right)\cdot E\left(m_{2},r_{2}\right)\right)\mathrm{mod}n^{2}=\left(m_{1}+m_{2}\right)\mathrm{mod}n\\ & D\left(E\left(m_{1},r_{1}\right)⊖E\left(m_{2},r_{2}\right)\right)\mathrm{mod}n^{2}=D\left(E\left(m_{1},r_{1}\right)\cdot E{\left(m_{2},r_{2}\right)}^{-1}\right)\mathrm{mod}n^{2}=\left(m_{1}-m_{2}\right)\mathrm{mod}n\\ & D\left(E{\left(m,r\right)}^{k}\right)\mathrm{mod}n^{2}=\left(k\cdot m\right)\mathrm{mod}n. \end{array} \end{array}$$ (1)

In the following context, we leave out the mod operation without confusion for easy exposition.

### Zero-knowledge proof

Zero-knowledge proof (ZKP) is one of the most popular tools in the recent cryptographic field. The fundamental notion of zero-knowledge was introduced by Goldwasser, Micali, and Rackoff in [28]. The most intriguing nature of ZKP is that the prover tries to convince the verifier about the validity of a statement without revealing any secret more beyond the statement. For example, from the commitment *Com* = *g*^*x*^ *h*^*r*^, in a zero-knowledge manner, the predicate (*x* = 1∨*x* = 0) can be proven by the proof of knowledge *POK*(*r*: *Com*/*g* = *h*^*r*^∨*Com* = *h*^*r*^).

### Garbled circuits

The seminal work of Yao’s garbled circuits was presented in [29]. The secure subtraction circuit (SecSub), the secure comparison circuit (SecCmp), and the secure minimum circuit (SecMin) were proposed in [26, 30]. We observe that the object value is obtained by recursively invoking a basic circuit (e.g., SUB, CMP, and MIN). Functionally, the secure subtraction circuit SecSub is used to subtract two *l*-bit integers *a* and *b* securely. In addition, the secure comparison circuit SecCmp is employed to compare two *l*-bit integers *a* and *b* efficiently. Moreover, the secure minimum circuit SecMin is constructed to find the minimum value from a list of values. For more details, we refer the readers to [26, 30].

### Partially blinded signature

The partially blinded signature was proposed in [31] and extended in [32]. The blinded signature allows a user to sign a message without revealing any information about the message. Original blind signatures cannot embed information (e.g., expire date). Hence, it creates an issue that when the previous signatures expire, the signer is to issue new public keys. Partially blinded signatures overcome this shortcoming in which the signer can embed the expire date in the signed message. In this way, signed information can be safely deleted once they are outdated. In [16], for liability attribution, the partially blinded signature was modified so that the user’s public identity was revealed if a user tried to use the same token more than once.

## Our scheme

Our procurement bidding system consists of three phases: the identity verification of suppliers (IdVfy), double-spending check (DSChk), and bid comparison (BidCmp). IdVfy is used by the PA to verify the identities of suppliers. In addition, DSChk is used by the PA to check whether there exist double-spending behaviors, i.e., whether a supplier submits the same bid more than once. Finally, BidCmp is employed by the PM to select securely the winning supplier with the bid closest to the average value of all the bids. Before we describe these phases in detail, we first introduce the setup process for our scheme.

### Setup

Let *κ* denote the system security parameter. Two large primes *p* and *q* are selected. Let *n* = *p* ⋅ *q*, $g,h,h_{1}\overset{\$}{\leftarrow }Z_{n^{2}}^{*}$, *s* = (*p* − 1) ⋅ (*q* − 1), and *w* = *g*^*s*^. We encode the system parameter as *params* = (*g*, *h*, *h*_1_, *w*) and the expiration time as *z* = *H*(*params*, *expiration*), where *H* denotes a secure hash function. The private key is *sk* = *s* and the public key is *pk* = (*g*, *h*, *h*_1_, *w*, *z*).

### IdVfy

First, the supplier computes a commitment $C=h^{r}h^{y_{i}}$ within its secret ID *y*_*i*_. Then the supplier needs to convince the PA that both *I* and *C* correspond to the same secret ID *y*_*i*_. To this end, along with *I* and *C*, the supplier sends to the PA the proof $POK\{\left(r,y_{i}\right):C=h^{r}h_{1}^{y_{i}}\wedge I=h_{1}^{y_{i}}\}$ of knowledge of *r* and *y*_*i*_. The identity verification process can be described in Algorithm 1.

**Algorithm 1**: IdVfy

**Require**: a random value $r\overset{\$}{\leftarrow }Z_{n^{2}}^{*}$, the secret identity ID *y*_*i*_

**Ensure**: True or False

 At Supplier:

1: Choose $\alpha ,\beta ,r\overset{\$}{\leftarrow }Z_{n^{2}}^{*}$;

2: Compute $C=h^{r}h_{1}^{y_{i}},I=h_{1}^{y_{i}},P_{1}=h^{\alpha }h_{1}^{\beta },P_{2}=h_{1}^{\beta }$;

3: Send *C*, *I*, *P*_1_, *P*_2_ to the PA;

 At the PA:

4: Send a challenge *e* to the supplier;

 At the Supplier:

5: Compute $z_{r}=\alpha -er,z_{y_{i}}=\beta -ey_{i}$;

6: Send $z_{r},z_{y_{i}}$ to the PA;

 At the PA:

7: **if** $P_{1}\overset{?}{=}C^{e}h^{z_{r}}h_{1}^{z_{y_{i}}}\wedge P_{2}\overset{?}{=}I^{e}h_{1}^{z_{y_{i}}}$ **then**

8:  Accept the proof;

9:  **return** True;

10: **else**

11:  Reject the proof;

12:  **return** False;

13: **end if**

The correctness of *P*_1_ and *P*_2_ in Algorithm 1 can be validated through Eq 2.

$$\begin{array}{c} \begin{array}{cc} P_{1} & =h^{\alpha }h_{1}^{\beta }=h^{er-er}h^{\alpha }h_{1}^{\beta }h_{1}^{ey_{i}-ey_{i}}=h^{z_{r}}h^{er}h_{1}^{z_{y_{i}}}h_{1}^{ey_{i}}=h^{z_{r}}h_{1}^{z_{y_{i}}}{\left(h^{r}h_{1}^{y_{i}}\right)}^{e}=C^{e}h^{z_{r}}h_{1}^{z_{y_{i}}}\\ P_{2} & =h_{1}^{\beta }=h_{1}^{\beta }h_{1}^{ey_{i}-ey_{i}}=h_{1}^{ey_{i}}h_{1}^{\beta -ey_{i}}=I^{e}h_{1}^{z_{y_{i}}}. \end{array} \end{array}$$ (2)

That completes the proof.

### DSChk

By following the work of [16], we customize a partially blinded signature to check the suppliers’ double-spending behaviors. First, the PM picks $r_{1}\overset{\$}{\leftarrow }Z_{n_{2}}^{*}$ and creates one-time tags $z_{1}=Cg^{r_{1}},z_{2}=\frac{z}{z_{1}}$. Then the supplier blinds *z*, *z*_1_, *z*_2_ into $\zeta =z^{\gamma },\zeta_{1}=z_{1}^{\gamma },\zeta_{2}=\frac{\zeta }{\zeta_{1}}$. Later on, the supplier selects $\tau \overset{\$}{\leftarrow }Z_{n_{2}}^{*}$ and computes *η* = *z*^*τ*^ which serves as a commitment. Then the PA proves in zero knowledge that it knows the secret values corresponding to *w* and *z*. Moreover, the ZK proof is converted to a signature *σ*. Then the supplier computes *ε*_1_, *μ*_1_ and performs the signature validity test. If the supplier exists double-spending behaviors, there will be two different *ε*_1_, *μ*_1_ and $\varepsilon_{1}^{\prime },\mu_{1}^{\prime }$ associated with the same identity *I*. Hence, the PA can compute $r=\frac{{\mu_{1}}^{\prime }-\mu_{1}}{\varepsilon_{1}-\varepsilon_{1}^{\prime }}$ and retrieve the public identity of the supplier as $I=I^{\prime \frac{1}{r}}$. The double-spending checking details are described in Algorithm 2 where a function *DleSpend* invokes Algorithm 3.

**Algorithm 2**: DSChk

**Require**: the encrypted bid 〈*b*_*i*_〉, the commitment *C*, the value *r* corresponding to *y*_*i*_, the public key *pk* = (*g*, *h*, *h*_1_, *w*, *z*), the private key *sk* = *s*, the list *L*;

**Ensure**: the double-spending public identity *I* or *NULL*;

At the PA:

1: Choose a random value $r_{1}\overset{\$}{\leftarrow }Z_{n^{2}}^{*}$, compute $z_{1}=Cg^{r_{1}},z_{2}=\frac{z}{z_{1}}$ and send *r*_1_ to the supplier;

 At the Supplier:

2: Choose $\gamma ,\tau \overset{\$}{\leftarrow }Z_{n^{2}}^{*}$ and compute $z_{1}=Cg^{r_{1}},\zeta =z^{\gamma },\zeta_{1}=z_{1}^{\gamma },\zeta_{2}=\frac{\zeta }{\zeta_{1}},\eta =z^{\tau }$;

 At the PA:

3: Choose $\mu ,r_{2},r_{3},r_{4},\overset{\$}{\leftarrow }Z_{n^{2}}^{*}$ and compute $a=g^{\mu },a_{1}=g^{r_{2}}z_{1}^{r_{4}},a_{2}=h^{r_{3}}z_{2}^{r_{4}}$;

4: Send *a*, *a*_1_, *a*_2_ to the supplier;

 At the supplier:

5: Choose $t_{1},t_{2},t_{3},t_{4},t_{5}\overset{\$}{\leftarrow }Z_{n^{2}}^{*}$ and compute $\alpha =ag^{t_{1}}w^{t_{2}},\alpha_{1}=a_{1}^{\gamma }g^{t_{3}}\zeta_{1}^{t_{4}},\alpha_{2}=a_{2}^{\gamma }h^{t_{5}}\zeta_{2}^{t_{4}},\varepsilon =H\left(\zeta ,\zeta_{1},\alpha ,\alpha_{1},\alpha_{2},\eta ,\langle b_{i}\rangle \right),\nu =\varepsilon -t_{2}-t_{4}$;

6: Send *ν* to the PA;

 At the PA:

7: Compute *c* = *ν* − *r*_4_, *r*_5_ = *μ* − *cs* and send *c*, *r*_2_, *r*_3_, *r*_4_, *r*_5_ to the supplier;

 At the supplier:

8: Choose $R\overset{\$}{\leftarrow }Z_{n^{2}}^{*}$, compute *ρ* = *r*_5_ + *t*_1_, *ω* = *c* + *t*_2_, *ρ*_1_ = *γr*_2_ + *t*_3_, *ρ*_2_ = *γr*_3_ + *t*_5_, *ω*_1_ = *r*_4_ + *t*_4_, *θ* = *τ* − *ω*_1_ *γ*, *σ* = (*ζ*, *ζ*_1_, *ρ*, *ω*, *ρ*_1_, *ρ*_2_, *ω*_1_, *θ*), *ε*_1_ = *H*(*σ*, *I*^*r*^, *R*), *ϕ* = *H*(*I*), *μ*_1_ = *ϕ* − *ε*_1_ *r*, *I*′ = *I*^*r*^, send (*ε*_1_, *ϕ*, *μ*_1_, *I*′) to the PA;

 At the PA:

9: **if** $\omega +\omega_{1}\overset{?}{=}H\left(\zeta ,\zeta_{1},g^{\rho }w^{\omega },g^{\rho_{1}}\zeta_{1}^{\omega_{1}},h^{\rho_{2}}\zeta_{2}^{\omega_{1}},z^{\mu_{1}}\zeta^{\varepsilon_{1}}\right)$ **then**

10: Set Γ = *DleSpend*((*ε*_1_, *ϕ*, *μ*_1_, *I*′), *L*);

11: **end if**

**Algorithm 3**: DleSpend

**Require**: the tuple (*ε*_1_, *ϕ*, *μ*_1_, *I*′), the list *L*;

**Ensure**: the double-spending public identity *I* or *NULL*;

 At the PA:

1: **for** *i* = 1 to *L*.*length* **do**

2:  Fetch *L*(*i*) into the tuple $\left(\varepsilon_{1}^{\prime },\phi^{\prime },\mu_{1}^{\prime },I^{\prime }\right)$;

3:  **if** *ϕ* = = *ϕ*′ **then**

4:   Compute $r=\frac{\mu_{1}^{\prime }-\mu_{1}}{\varepsilon_{1}-\varepsilon_{1}^{\prime }}$;

5:   Compute $I=I^{\prime \frac{1}{r}}$;

6:   **return** *I*;

7:  **else**

8:   Append the tuple (*ε*_1_, *ϕ*, *μ*_1_, *I*′) into *L*;

9:   **return** *NULL*;

10:  **end if**

11: **end for**

### BidCmp

**Algorithm 4**: BidCmp

**Require**: the encrypted bids 〈*b*_1_〉, 〈*b*_2_〉, …, 〈*b*_*m*_〉, the number of suppliers *m*, the key pair (*sk*, *pk*);

**Ensure**: The index *i*_*w*_ of the supplier with the bid closest to the average value of all the bids;

 At the PM:

1: Choose *m* *k*-bit random values *r*_1_, *r*_2_, …, *r*_*m*_;

2: Initialize *B* = 1;

3: **for** *i* = 1 to *m* **do**

4:  Compute *B* = *B* ⋅ 〈*b*_*i*_〉;

5: **end for**

6: **for** *i* = 1 to *m* **do**

7:  Compute ${B_{i}}^{\prime }={\langle b_{i}\rangle }^{m}⊖B\cdot \langle r_{i}\rangle$;

8: **end for**

9: Send ${B_{1}}^{\prime },{B_{2}}^{\prime }\ldots ,{B_{m}}^{\prime }$ to the PA;

 At the PA:

10: **for** *i* = 1 to *m* **do**

11:  Decrypt ${B_{i}}^{\prime }$ to get $B_{i}=\left(mb_{i}-\sum_{i=1}^{m}b_{i}+r_{i}\right)$;

12:  Compute $B_{i}B_{i}={\left(mb_{i}-\sum_{i=1}^{m}b_{i}+r_{i}\right)}^{2}$;

13:  Re-encrypt *B*_*i*_ *B*_*i*_;

14: **end for**

15: Send 〈*B*_1_ *B*_1_〉, 〈*B*_2_ *B*_2_〉, …, 〈*B*_*m*_ *B*_*m*_〉 to the PM;

 At the PM:

16: **for** *i* = 1 to *m* **do**

17:  Compute $B_{i}^{*}=\langle B_{i}B_{i}\rangle ⊖{\left({\langle b_{i}\rangle }^{m}⊖B\right)}^{2r_{i}}⊖\langle r_{i}^{2}\rangle$;

18: **end for**

**Fig 2:**
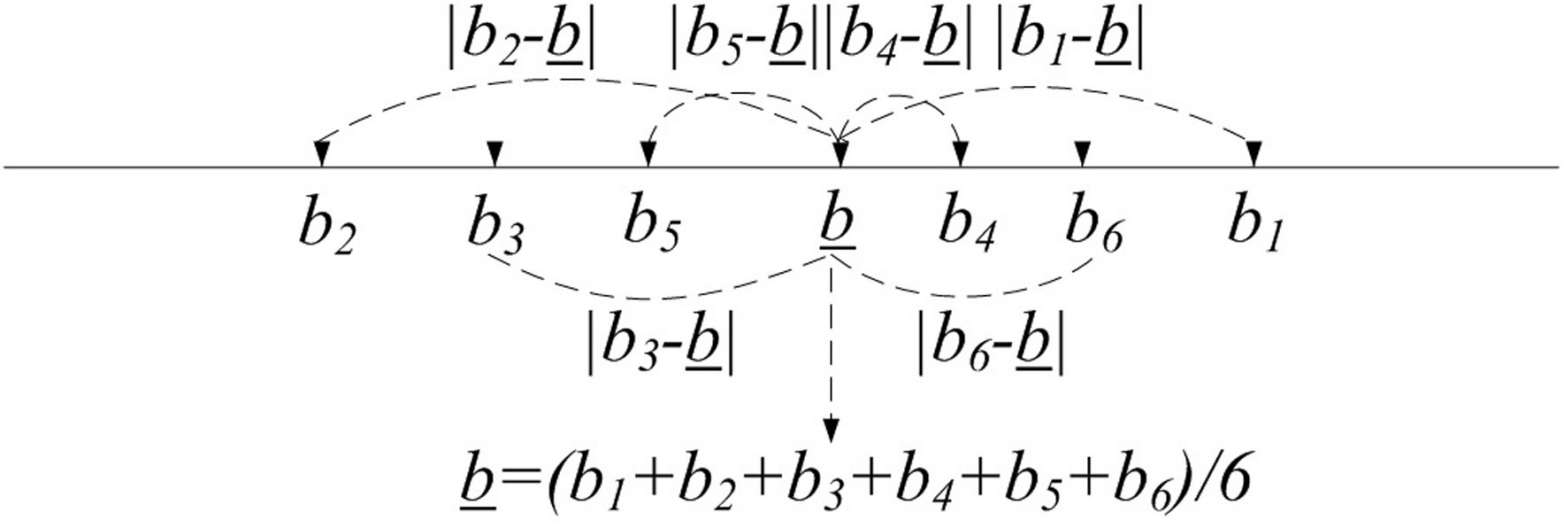
The high-level structure of average procurement bidding system.

19: Set *i*_*w*_ = **SecMin** $\left(B_{1}^{*},B_{2}^{*},\ldots ,B_{m}^{*}\right)$;

20: **return** *i*_*w*_;

We assume the number of suppliers *m* equals to 6, then the high-level structure of average procurement bidding system is described in Fig 2. In order to find the supplier with the bid closest to the average value of all the bids securely, first, for each supplier, we should measure the distance between the bid *b*_*i*_ and the average bid $\underline{b}$, then we have to find the minimum absolute value of these distances. That is, we devote to find the minimum value of $|b_{1}-\underline{b}\left|,\right|b_{2}-\underline{b}\left|,\ldots ,\right|b_{m}-\underline{b}|$. Moreover, with the consideration of privacy preservation, the minimum value selection of $|b_{1}-\underline{b}\left|,\right|b_{2}-\underline{b}\left|,\ldots ,\right|b_{m}-\underline{b}|$ is desired to achieve on the ciphertexts. However, it is hard to compute the minimum value from $|b_{1}-\underline{b}\left|,\right|b_{2}-\underline{b}\left|,\ldots ,\right|b_{m}-\underline{b}|$ on the ciphertexts directly. Therefore, we should find out another candidate way. Since $\underline{b}=\frac{1}{m}\sum_{i=1}^{m}b_{i}$ and $B=\sum_{i=1}^{m}b_{i}$, we can transform the work to compute the minimum value from 〈(*mb*_1_ − *B*)^2^〉, 〈(*mb*_2_ − *B*)^2^〉, …, 〈(*mb*_*m*_ − *B*)^2^〉 where 〈⋅〉 donates the ciphertext form. Algorithm 4 is designed to compute the supplier with the bid closest to the average value of all the bids.

We describe Algorithm 4 in detail. First, we introduce the input values for BidCmp. The PA keeps the private key *sk* and the PM holds the encrypted bids 〈*b*_1_〉, 〈*b*_2_〉, …, 〈*b*_*m*_〉. The PM selects *m* random values *r*_1_, *r*_2_, …, *r*_*m*_. Then the PM calculates ${B_{1}}^{\prime },{B_{2}}^{\prime }\ldots ,{B_{m}}^{\prime }$ and sends them to the PA. The PA computes and sends 〈*B*_1_ *B*_1_〉, 〈*B*_2_ *B*_2_〉, …, 〈*B*_*m*_ *B*_*m*_〉 to the PM. By using Paillier cryptosystem’s homomorphic properties and the secure minimum value circuit SecMin. The PM chooses the winning supplier *i*_*w*_ with the bid closest to the average value of all the bids.

## Performance analysis

### Security analysis

The phases IdVfy and DSChk are designed to support the double-spending checking functionality in our scheme. The main cryptographic tools in these phases are zero-knowledge proof and the partially blind signature scheme. Their security in these two phases has been proven in [32, 33], respectively. Hence, our study focuses on the demonstration of the security of BidCmp under the semi-honest model.

The high level of the algorithm is described before providing a formal definition. Bob generates a key pair, (*sk*, *pk*), and sends the public key, *pk*, to Alice. Alice encrypts the data and masks them with random values by leveraging the homomorphic properties of a specific cryptosystem. Then, Alice sends the masked encrypted data to Bob. Then, Bob decrypts the data and performs some operations on the decrypted data. Subsequently, Bob re-encrypts the result and sends it to Alice. The privacy of Bob can be guaranteed by the semantic security of the adopted cryptosystem. Moreover, Bob does not have any knowledge of Alice’s privacy during the process. Similar to the works in [20, 21, 34], the formal security of an algorithm under the semi-honest model is presented as follows.

**Definition 1 (Security)**: *An algorithm* Π *has two parties Alice (resp. Bob) and computes f*^*A*^(*x*, *y*) (*resp*. *f*^*B*^(*x*, *y*)), *where* (*x*, *y*) *are inputs of Alice and Bob, respectively. Let* $V_{\Pi }^{A}\left(x,y\right)$ (*resp*. $V_{\Pi }^{B}\left(x,y\right)$) *denote as Alice’s (resp. Bob’s) view during executing* Π *on the input of* (*x*, *y*). *Moreover*, $\left(x,r_{A},{\left\{m_{i}\right\}}_{i=1}^{t}\right)$ (*resp*. $\left(y,r_{B},{\left\{m_{i}\right\}}_{i=1}^{t}\right)$) *are Alice’s (resp. Bob’s) input, randomly selected values, and passed messages between the parties. Then the algorithm* Π *is secure against semi-honest adversaries if there are probabilistic polynomial time (PPT) simulators S*_1_ *and S*_2_ *that make* Eq 3 *hold. Recall that the symbol* ≈_*c*_ *means computational indistinguishability*.

$$\begin{array}{c} \begin{array}{cc} & \left(S_{1}\left(x,f^{A}\left(x,y\right)\right),f^{B}\left(x,y\right)\right)\approx_{c}\left(V_{A}^{\Pi }\left(x,y\right),O_{B}^{\Pi }\left(x,y\right)\right),\\ \; & \left(S_{2}\left(y,f^{B}\left(x,y\right)\right),f^{A}\left(x,y\right)\right)\approx_{c}\left(V_{B}^{\Pi }\left(x,y\right),O_{A}^{\Pi }\left(x,y\right)\right). \end{array} \end{array}$$ (3)

Based on Definition 1, we can obtain the following Lemma 1.

**Lemma 1**: *Assume Bob generate the key pair* (*pk*, *sk*) *for the homomorphic cryptographic system and issue the public key pk to Alice. Then Alice and Bob run the algorithm* Π. *All the ciphertexts transmitted from Alice to Bob are uniformly distributed and independent of Alice’s inputs. And all the messages transmitted from Bob to Alice are encrypted by the cryptographic system. Therefore, the algorithm* Π *is secure against semi-honest adversaries*.

**Proof**. To prove Lemma 1, we consider simulators in two different cases depending on which party is corrupted by the adversary. In the first case, Alice is corrupted and in the second case, Bob is corrupted. Moreover, in each case, finally we can infer that Eq 3 holds. Hence, we conclude that the algorithm *Π* is secure against semi-honest adversaries. In [9], we can see more details.

**Theorem 1**: *BidCmp* (*Algorithm 4) is secure against semi-honest adversaries*.

**Proof**. In Algorithm 4, messages are exchanged between the PM and the PA. By using random values, messages are masked and sent from the PM to the PA including ${B_{1}}^{\prime },{B_{2}}^{\prime }\ldots ,{B_{m}}^{\prime }$, which are uniformly distributed in the ciphertext space $Z_{n^{2}}$. In addition, messages 〈*B*_1_ *B*_1_〉, 〈*B*_2_ *B*_2_〉, …, 〈*B*_*m*_ *B*_*m*_〉, which are encrypted by the semantically secure Paillier cryptosystem, are sent from the PA to the PM. Moreover, SecMin is a direction application of Yao’s garbled circuit and its security has been demonstrated in [35]. Therefore, based on Lemma 1 and sequential composition theory [36]. BidCmp is secure against semi-honest adversaries.

**Theorem 2**: *Since the security of the above algorithms are secure, then our scheme PSPAB is secure against semi-honest adversaries*.

**Proof**. The private information (e.g., bids) is well-protected by the cryptographic system. Besides, BidCmp is secure against semi-honest adversaries. Hence, no information about secret bids is disclosed to each other party. That means, our scheme PSPAB is secure against semi-honest adversaries.

### 0.1 Efficiency analysis

We measure the individual computation time and communication cost in each phase (IdVfy, DSChk, and BidCmp) of PSPAB, and then measure the corresponding overall values.

#### IdVfy

The computation complexity and communication complexity in this phase mainly originate from Algorithm 1. This phase is executed between the suppliers and the PA to verify the identities of the suppliers. The computation complexity for each supplier involves the operations of identity commitment, proof generation, and proof verification. The communication complexity for each supplier involves an exchange of 7 values between the suppliers and the PA. The number of exchanged values is constant for each supplier. Therefore, the computation complexity and communication complexity are both *O*(*m*).

#### DSChk

The computation complexity and communication complexity in this phase mainly originate from Algorithm 2. This phase is computed between the suppliers and the PA to check whether there are double-spending behaviors. The computation complexity for each supplier involves the same commit-challenge-verify process between the suppliers and the PA. Besides, the communication complexity for each supplier involves an exchange of 14 values between the suppliers and the PA. Hence, the computation complexity and communication complexity are both *O*(*m*).

#### BidCmp

The computation complexity and communication complexity in this phase mainly originate from Algorithm 4. This phase is executed between the PM and the PA to seek out the supplier with the bid closest to the average of all the bids by the SecMin garbled circuit. Besides, it involves an exchange of 2*m* values including ${B_{1}}^{\prime },{B_{2}}^{\prime }\ldots ,{B_{m}}^{\prime }$ and 〈*B*_1_ *B*_1_〉, 〈*B*_2_ *B*_2_〉, …, 〈*B*_*m*_ *B*_*m*_〉 between the PM and the PA. Therefore, the computation complexity and communication complexity are *O*(*m*).

In sum, with a combination of three phases, the computation complexity and communication complexity are both *O*(*m*).

## Experimental results

The core cryptographic operations are prototypically implemented using Java to demonstrate the feasibility of PSPAB. All the experiments were developed by a laptop with Intel i7-6560U CPU, 2.20 GHz clock. We install Java Development Kit (JDK) of the version 1.8.0 191 and configure Eclipse IDE for Java Developers of the version 2018-12 (4.10.0) under Windows Operating System 7. Moreover, we use the provided packages, such as java.security, java.math, and java.util. The garbled circuits use 80-bit wire labels and Paillier cryptosystem uses the 1024-bit modulus. The system parameters are set as follows. The number of suppliers, *m*, varies from 200 to 1000. The bit length, *l*, of the bids spans from 30 to 70 and the bit length, *k*, of the masked random values is 30 bits longer than *l*. The default values of the number of suppliers, *m*, and the bit length, *l*, are set as 200 and 30, respectively. All experimental results including computation time and communication cost are on an average of 10 runs. In this paper, we intend to answer the following questions.

1. How do different numbers of suppliers affect the computation time and communication cost?
2. How do different bit lengths affect the computation time and communication cost?
3. How efficient is PSPAB in terms of the computation time and communication cost, in comparison with the state-of-the-art works?

We are mainly concerned with two metrics in the performance evaluations.

1. Computation time. The overall processing time is derived from the following phases of PSPAB. It consists of the total computation time at the PA’s side and the suppliers’ side in IdVfy and DSChk, and the total computation time at the PM’s side and the PA’s side in BidCmp.
2. Communication cost. The overall communication cost in different phases consists of the communication costs between the PA and the suppliers in IdVfy and DSChk, and the communication cost between the PM and the PA in BidCmp. We use a system function “System.nanoTime” in Java to obtain the computation time. For example, we present a pseudo code of computation time for BidCmp in Algorithm 5. Note that 10 computation times for BidCmp are measured, and then, the average computation time is obtained for better precision.

**Algorithm 5**: Computation time for BidCmp

1: Initialize starttime = 0; endtime = 0; avgtime = 0; loopcount = 10;

2: **for** *i* = 1 to loopcount **do**

3:  starttime = System.nanoTime();

4:  BidCmp;

5:  endtime = System.nanoTime();

6:  avgtime = avgtime + (endtime—starttime);

7: **end for**

**Fig 3:**
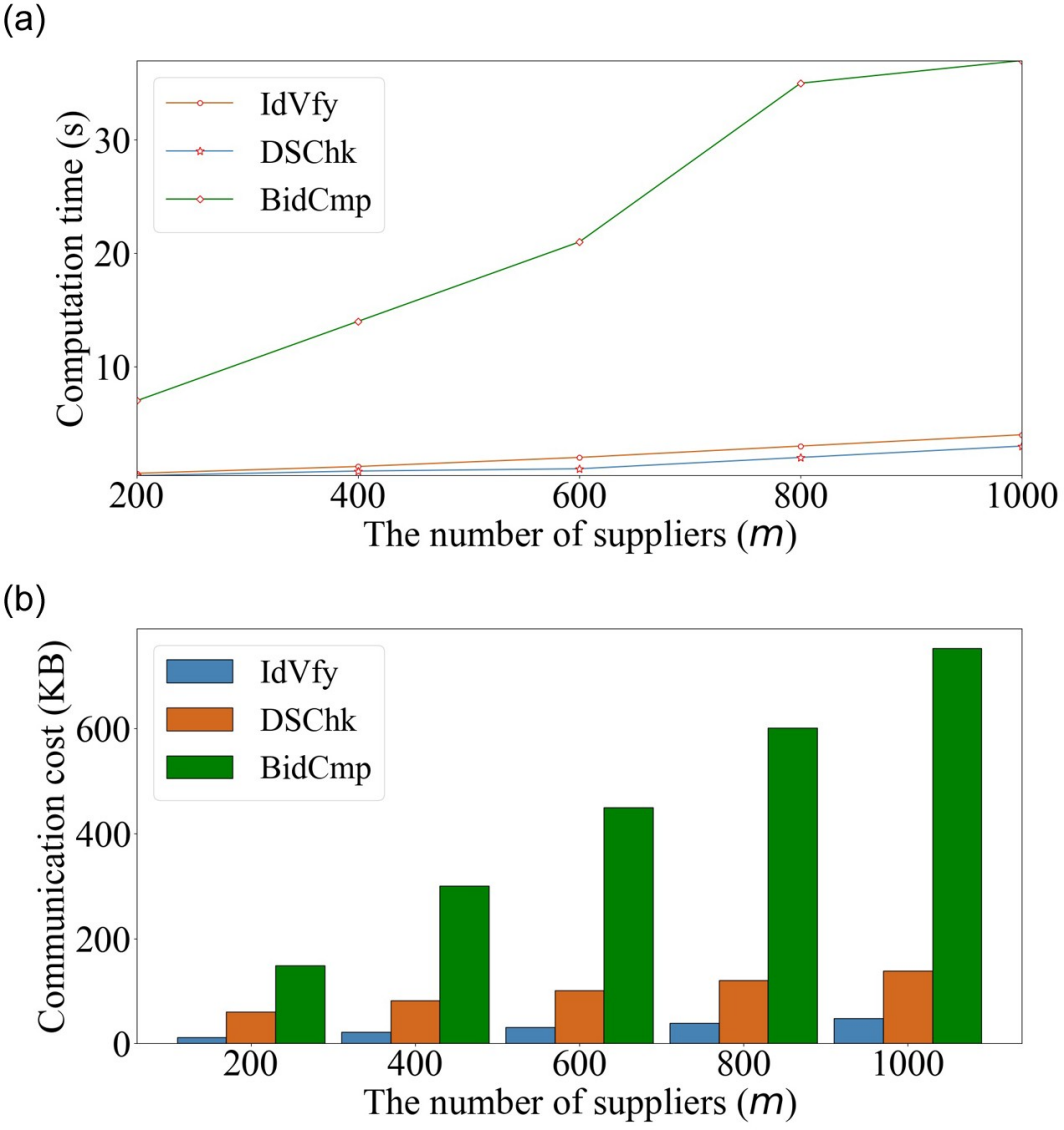
Computation time and communication cost in different phases.

**Fig 4:**
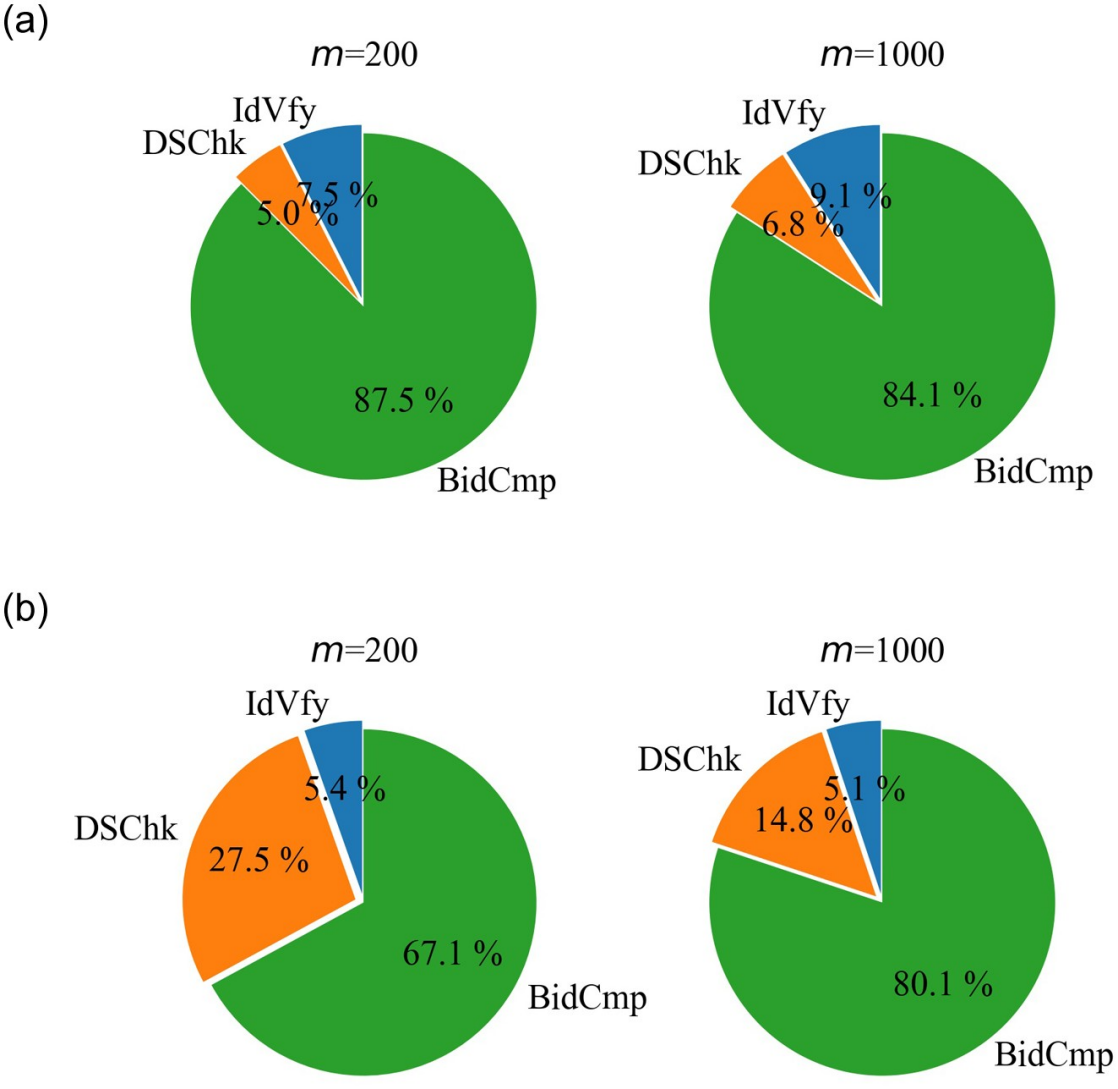
The proportion of computation time and communication cost in different phases.

8: avgtime = avgtime / loopcount;

9: **return** avgtime;

We evaluate the performance of our scheme including three phases IdVfy, DSChk, and BidCmp in Figs 3 and 4. For *m* = 200, the computation times in different phases and their percentages are as follows: 0.6 s in IdVfy accounting for 7.5%, 0.4 s in DSChk accounting for 5%, and 7 s in BidCmp accounting for 87.5%. In addition, the communication costs in different phases and their percentages are as follows: 12KB in IdVfy accounting for 5.4%, 61KB in DSChk accounting for 27.5%, and 149KB in BidCmp accounting for 67.1%. For *m* = 1000, the computation times in different phases and their percentages are in the following: 4s in IdVfy accounting for 9.1%, 3s in DSChk accounting for 6.8%, and 37s in BidCmp accounting for 84.1%. In addition, the communication costs in different phases and their percentages are as follows: 48KB in IdVfy accounting for 5.1%, 139KB in DSChk accounting for 14.8%, and 753KB in BidCmp accounting for 80.1%. It is observed that BidCmp accounts for over 80% and 60% of the overall computation time and communication cost, respectively. Therefore, we consider BidCmp as the most time-consuming and storage-consuming phase of PSPAB. This is because the multiple homomorphic operations used in BidCmp are more computationally intensive than the commitment operation in IdVfy and the partially blind signature operation in DSChk.

**Fig 5:**
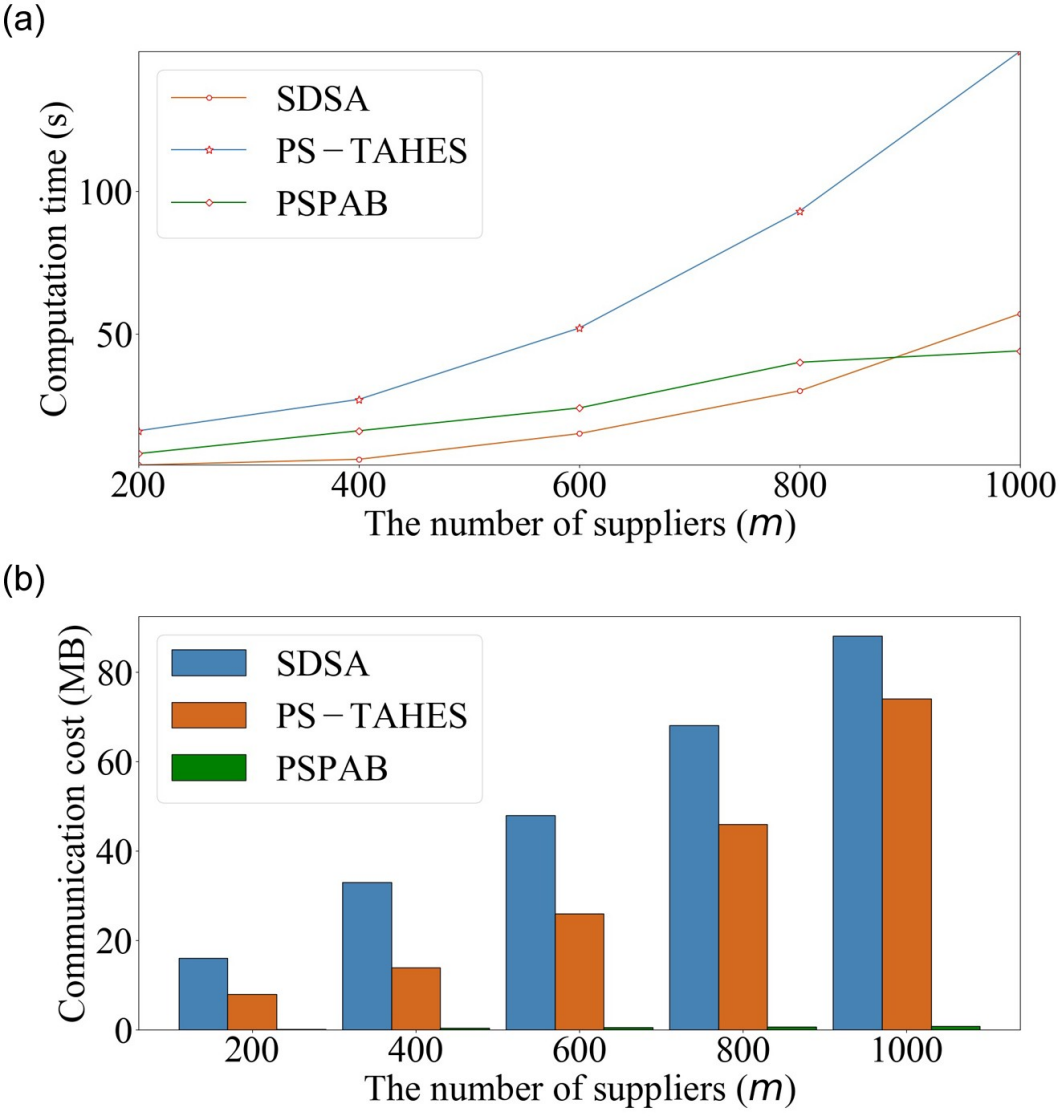
Performance comparisons in computation time and communication cost.

We compare our scheme with two state-of-the-art works, i.e., SDSA [8] and PS-TAHES [9], in Fig 5 for a well-rounded performance evaluation. The computation time and communication cost increase with the number of suppliers, *m*. We observe that the increase rate of these parameters for PSPAB is more moderate compared with those of SDSA and PS-TAHES. For *m* = 1000, the computation time and communication cost of PSPAB are 44 s and 940KB, respectively. The computation time is shorter than those of PS-TAHES (149 s, 3.4 × faster) and SDSA (57 s, 1.3 × faster). The communication cost is also lower than those of PS-TAHES (74MB, 82 × smaller) and SDSA (88MB, 97 × smaller). We can see more details in Tables 3 and 4. The reason for this performance improvement is that our framework is general and efficient, which avoids computation-intensive operations; moreover, it applies lightweight cryptographic tools to provide bid privacy protection.

**Table 3:** Computation time with previous works (s).

Scheme	*m* = 200	*m* = 400	*m* = 600	*m* = 800	*m* = 1000
SDSA	4	6	15	30	57
PS-TAHES	16	27	52	93	149
PSPAB	8	16	24	40	44

**Table 4:** Communication cost with previous works (MB).

Scheme	*m* = 200	*m* = 400	*m* = 600	*m* = 800	*m* = 1000
SDSA	16	33	48	68	88
PS-TAHES	8	14	26	46	74
PSPAB	0.2	0.4	0.6	0.7	0.9

**Fig 6:**
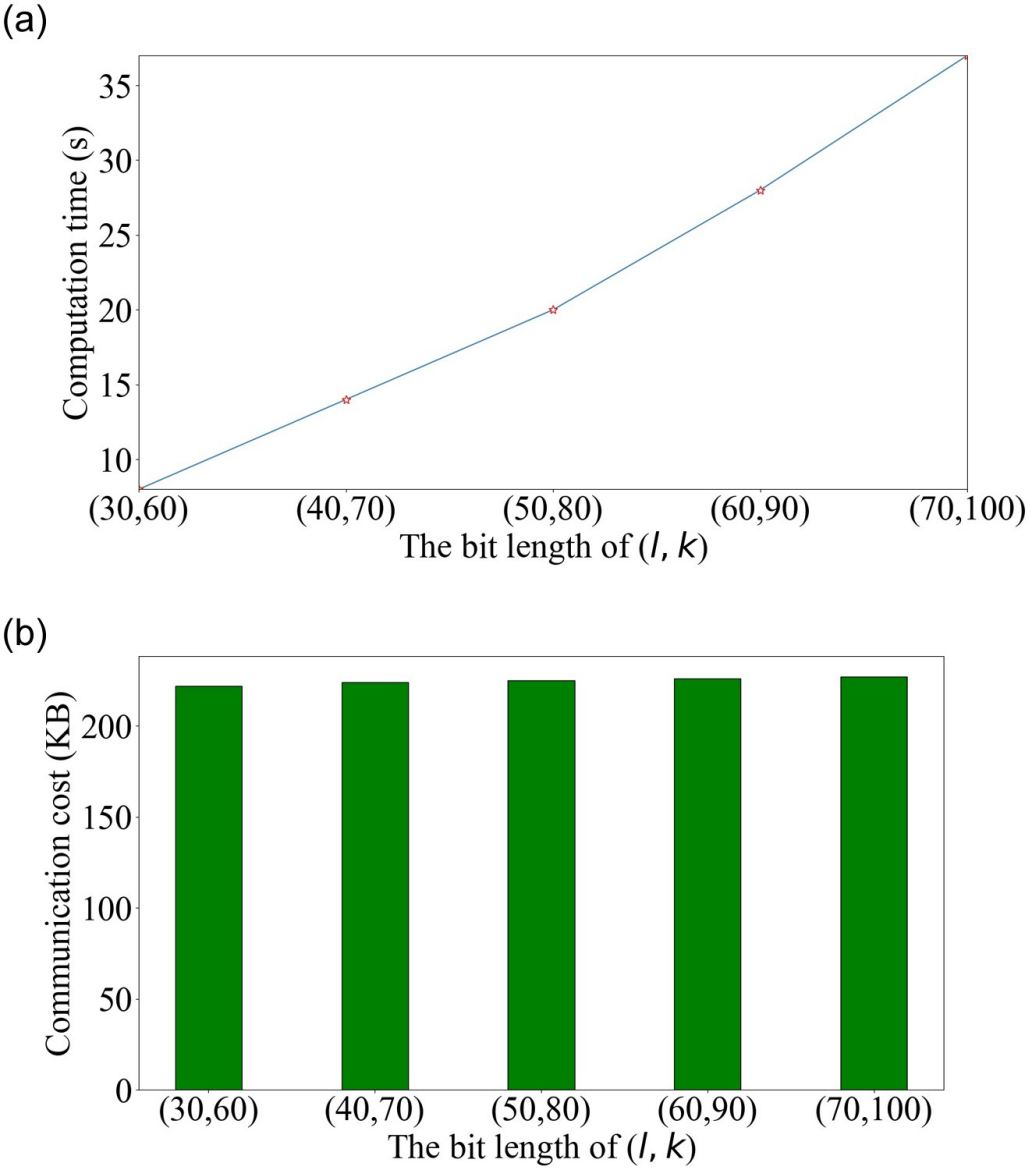
Computation time and communication cost with the bit lengths of (*l*, *k*).

In Fig 6, we fix *m* to 200 and vary the bit length, *l*, of bids from 30 to 70. Accordingly, the bit length, *k*, of the randomly masked values changes from 60 to 100, which provides the statistical security of 2^*l*−*k*^. We observe that the computation time increases with *l*, as it affects the execution time of the bid operations of PSPAB, e.g., secure bid comparison in BidCmp. However, as analyzed in the previous section, the number of exchanged values between the supplier and the PA in different phases remains constant for each supplier. Therefore, the communication cost almost remains constant with the increase in *l*.

## Conclusion and future work

In this paper, we proposed a lightweight average procurement bidding system (PSPAB) with the double-spending checking functionality. That is, we designed a series of secure basic operations by leveraging lightweight cryptographic primitives such as the Paillier cryptosystem, garbled circuits, and partially blind signature. Then, security analysis and performance analysis were performed. Finally, we compared PSPAB with two state-of-the-art works, SDSA and PS-TAHES, to demonstrate its superiority. Under the same system parameters, the computation time of PSPAB is 3.4 × faster and 1.3 × faster than PS-TAHES and SDSA, respectively. Besides, the communication cost is 82 × smaller and 97 × smaller than PS-TAHES and SDSA, respectively.

Our design can be further enhanced in the future. First, rather than the partially blind signature, a more efficient cryptographic tool can be used to enable double checking. Second, the latest multi-party comparison studies can be employed to make our scheme more efficient. Finally, we can make our scheme more widely applicable to other procurement bidding systems, such as the closest pretender bidding system.

## References

[1] Chang WS, Chen B, Salmon T. An investigation of the average bid mechanism for procurement auctions. Management Science. 2015; 61(6):1237–1254. 10.1287/mnsc.2013.1893

[2] Bianchi M, Spagnolo G, Albano GL. Bid average methods in procurement. Rivista di Politica Economica. 2006; 96:41–62.

[3] Decarolis F. Comparing public procurement auctions. International Economic Review. 2018; 59(2):391–419. 10.1111/iere.12274

[4] Conley T, Decarolis F. Detecting bidders groups in collusive auctions?. American Economic Journal: Microeconomics. 2016; 8:1–38.

[5] Lei M, Yin Z, Li S, Li H. Detecting the collusive bidding behavior in below average bid auction. In: 2017 13th International Conference on Natural Computation, Fuzzy Systems and Knowledge Discovery (ICNC-FSKD). IEEE; 2017. p. 1720-1727.

[6] Galal HS, Youssef AM. Verifiable sealed-bid auction on the ethereum blockchain In: Financial Cryptography and Data Security. Springer Berlin Heidelberg; 2019 p. 265–278.

[7] Wu S, Chen Y, Wang Q, Li M, Wang C, Luo X. CReam: A smart contract enabled collusion-resistant e-Auction. IEEE Transactions on Information Forensics and Security. 2019; 14(7):1687–1701. 10.1109/TIFS.2018.2883275

[8] Chen Z, Wei X, Zhong H, Cui J, Xu Y, Zhang S. Secure, efficient and practical double spectrum auction. In: 2017 IEEE/ACM 25th International Symposium on Quality of Service (IWQoS). IEEE/ACM; 2017. p. 1–6.

[9] Wang Q, Huang J, Chen Y, Tian X, Zhang Q. Privacy-preserving and truthful double auction for heterogeneous spectrum. IEEE/ACM Transactions on Networking. 2019; 27(2):848–861. 10.1109/TNET.2019.2903879

[10] Karame G, Androulaki E, Capkun S. Two bitcoins at the price of one? Double-spending attacks on fast payments in bitcoin. IACR Cryptol. ePrint Arch. 2012; 2012(248).

[11] Rosenfeld M. Analysis of hashrate-based double spending. arXiv preprint arXiv:1402.2009. 2014.

[12] Everaere P, Simplot-Ryl I, Traoré, I. Double spending protection for e-Cash based on risk management. In: Proceedings of the 13th International Conference on Information Security. Springer-Verlag; 2010. p. 394–408.

[13] Karame GO, Androulaki E, Roeschlin M, Gervais A, Čapkun S. Misbehavior in bitcoin: A study of double-spending and accountability. Acm Transactions on Information & System Security. 2015; 18(1): 1–32.

[14] Milutinovic M, Dacosta I, Put A, Decker BD. uCentive: An efficient, anonymous and unlinkable incentives scheme. In: 2015 IEEE Trustcom/BigDataSE/ISPA. IEEE; 2015. p. 588–595.

[15] Jager T, Rupp A. Black-box accumulation: Collecting incentives in a privacy-preserving way. In: Proceedings on Privacy Enhancing Technologies. Sciendo; 2016. p. 62–82.

[16] Dimitriou T, Giannetsos T, Chen L. REWARDS: Privacy-preserving rewarding and incentive schemes for the smart electricity grid and other loyalty systems. Computer Communications. 2019; 137: 1–14. 10.1016/j.comcom.2019.01.009

[17] Huang Q, Gui Y, Wu F, Chen G, Zhang Q. A general privacy-preserving auction mechanism for secondary spectrum markets. IEEE/ACM Transactions on Networking. 2016; 24(3):1881–1893. 10.1109/TNET.2015.2434217

[18] Blass EO, Kerschbaum F. Strain: A secure auction for blockchains In: Computer Security. Springer International Publishing; 2018 p. 87–110.

[19] Ma J, Qi B, Lv K. Fully private auctions for the highest bid. In: Proceedings of the ACM Turing Celebration Conference—China. ACM; 2019. p. 64:1–64:6.

[20] Chen Z, Huang L, Li L, Yang W, Miao H, Tian M, et al. PS-TRUST: Provably secure solution for truthful double spectrum auctions. In: IEEE INFOCOM 2014—IEEE Conference on Computer Communications. IEEE; 2014. p. 1249–1257.

[21] Wang Q, Huang J, Chen Y, Wang C, Xiao F, Luo X. *PROST*: Privacy-preserving and truthful online double auction for spectrum allocation IEEE Transactions on Information Forensics and Security. 2019; 14(2):374–386. 10.1109/TIFS.2018.2850330

[22] Chen Y, Tian X, Wang Q, Li M, Du M, Li Q. ARMOR: A secure combinatorial auction for heterogeneous spectrum. IEEE Transactions on Mobile Computing. 2019;18(10):2270–2284. 10.1109/TMC.2018.2875910

[23] Chen Z, Huang L, Chen L. ITSEC: An information-theoretically secure framework for truthful spectrum auctions. In: 2015 IEEE Conference on Computer Communications (INFOCOM). IEEE; 2015. p. 2065-2073.

[24] Goldreich O. Foundations of cryptography: vol. 2, chapter general cryptographic protocols. Cambridge university press; 2004.

[25] Lindell Y. How to simulate it—A tutorial on the simulation proof technique. In: Tutorials on the Foundations of Cryptography: Dedicated to Oded Goldreich. Springer International Publishing; 2017 p. 277–346. 10.1007/978-3-319-57048-8_6

[26] Goldreich O. Foundations of cryptography: volume 2, basic applications. Cambridge university press; 2009.

[27] Paillier P. Public-key cryptosystems based on composite degree residuosity classes In: Advances in Cryptology — EUROCRYPT’99. Springer Berlin Heidelberg; 1999 p. 223–238.

[28] Goldwasser S, Micali S, Rackoff C. The knowledge complexity of interactive proof systems. SIAM Journal on Computing. 1989; 18(1):186–208. 10.1137/0218012

[29] Yao AC. Protocols for secure computations. In: 23rd Annual Symposium on Foundations of Computer Science (sfcs 1982). IEEE; 1982. p. 160–164.

[30] Goldreich O Foundations of cryptography: volume 1, basic tools. Cambridge university press; 2007.

[31] Abe M. A secure three-move blind signature scheme for polynomially many signatures In: Advances in Cryptology — EUROCRYPT 2001. Springer Berlin Heidelberg; 2001 p. 136–151.

[32] Baldimtsi F, Lysyanskaya A. Anonymous credentials light. In: Proceedings of the 2013 ACM SIGSAC Conference on Computer and Communications Security. ACM; 2013. p. 1087–1098.

[33] Pedersen TP. Non-interactive and information-theoretic secure verifiable secret sharing In: Advances in Cryptology — CRYPTO’91. Springer Berlin Heidelberg; 1992 p. 129–140.

[34] Wang S, Xu P, Xu X, Tang S, Li X, Liu X. TODA: Truthful online double auction for spectrum allocation in wireless networks. In: 2010 IEEE Symposium on New Frontiers in Dynamic Spectrum (DySPAN). IEEE; 2010. p. 1–10.

[35] Lindell Y, Pinkas B. A proof of security of Yao’s protocol for two-party computation. Journal of Cryptology. 2009; 22(2):161–188. 10.1007/s00145-008-9036-8

[36] Hazay C, Lindell Y. Efficient secure two-party protocols: techniques and constructions. Springer Science & Business Media; 2010.

